# Urinary miRNA-27b-3p and miRNA-1228-3p correlate with the progression of Kidney Fibrosis in Diabetic Nephropathy

**DOI:** 10.1038/s41598-019-47778-1

**Published:** 2019-08-06

**Authors:** Francesca Conserva, Mariagrazia Barozzino, Francesco Pesce, Chiara Divella, Annarita Oranger, Massimo Papale, Fabio Sallustio, Simona Simone, Luigi Laviola, Francesco Giorgino, Anna Gallone, Paola Pontrelli, Loreto Gesualdo

**Affiliations:** 1 Department of Emergency and Organ Transplantation - Nephrology Unit, University of Bari Aldo Moro, Bari, Italy; 2 Department of Emergency and Organ Transplantation – Section of Internal Medicine, Endocrinology, Andrology and Metabolic Disease, University of Bari Aldo Moro, Bari, Italy; 3 Department of Basic Medical Sciences, Neurosciences and Sense Organs, University of Bari Aldo Moro, Bari, Italy

**Keywords:** Renal fibrosis, Molecular medicine

## Abstract

Diabetic Nephropathy (DN) is a chronic complication of diabetes and the primary cause of end stage renal disease. Differential diagnosis for DN requires invasive histological investigation, thus there is need for non-invasive biomarkers to discriminate among different histological lesions in diabetic patients. With the aim to identify a pattern of differentially expressed miRNAs in kidney biopsies of DN patients, we assayed miRNA expression in kidney biopsies from DN patients, diabetic patients with membranous nephropathy and patients with normal histology. Nine miRNAs were differentially expressed among the three groups, and 2 miRNAs (miR-27b-3p and miR-1228-3p) showed interaction with an ubiquitin-conjugating E2 enzyme variant (UBE2v1). UBE2v1 mediates the formation of lysine 63-linked ubiquitin chains, a mechanism we previously showed as involved in DN kidney fibrosis. Both miRNAs were validated as down-regulated in biopsies and urines of DN patients, possibly affected by DNA methylation. Interestingly, the urinary levels of both miRNAs could also discriminate among different degrees of renal fibrosis. Finally, we showed that the combined urinary expression of both miRNAs was also able to discriminate DN patients from other glomerulonephritides in diabetic patients. In conclusion we identified two miRNAs potentially useful as candidate biomarkers of tubular-interstitial fibrosis in diabetic patients with DN.

## Introduction

Diabetes mellitus (DM) is a dysmetabolic disease estimated to affect over 400 million of people by 2030^1,2^. The constantly growing prevalence of DM, along with the present impossibility to either predict and effectively treat diabetic kidney disease will become a major economic challenge in the near future.

Epidemiological studies show that one third of patients with DM develops diabetic nephropathy (DN) while other diabetic patients develop a non-diabetic renal disease (NDRD) such as membranous nephropathy (MN), IgA nephropathy, focal segmental glomerulosclerosis (FSGF), etc. Importantly, NDRDs may take place either alone or superimposed on DN^3^.

Currently, DN is routinely diagnosed on the basis of clinical findings such as albuminuria, ranging from micro- (30–300 mg/d) to macro-albuminuria (≥300 mg/d), and reduced glomerular filtration rate. Notably, these signs per se are not distinctive of DN as they also occur in patients with different forms of renal damage. Through the analysis of 2322 patients, Liang *et al*. concluded that the absence of diabetic retinopathy, shorter duration of diabetes, lower glycated hemoglobin (HbA1c) and lower blood pressure may be useful to identify DN patients among other classes of renal damage in type 2 diabetes^4^. In a recently published meta-analysis, our group observed that, among diabetic patients with biopsy-proven diagnosis, the prevalence of DN, NDRD and mixed forms ranges from 6.5 to 94%, 3 to 82.9% and 4 to 45.5% respectively^5^. Clinical judgment alone can lead to wrong diagnoses and delay the establishment of adequate therapies. Indeed, renal biopsy remains the gold standard for a correct diagnosis of DN^6^, but due to the possible procedural complications it is often confined to cases showing an atypical clinical presentation^7,8^. The differential diagnosis between DN and NDRD is thus still challenging. There is an urgent need for specific, rapid, non-invasive and inexpensive biomarkers to discriminate NDRD from DN forms; this would help clinicians in making an accurate diagnosis and set the most appropriate therapy^9^.

In this scenario, microRNA may represent ideal candidate biomarkers^10^. MicroRNAs (miRNAs) act as translational repressors of specific messenger RNAs (mRNAs) through perfect- or partial-match binding^11^, are highly tissue-specific and can circulate in body fluids either associated with RNA binding proteins or in exosome-like lipid vesicles, protected from degradation^12^. Several mechanisms can modulate miRNA expression in various organs and during disease.

Recent research explored the urinary miRNome of type 1 DN^13^. Rudnicki *et al*. performed miRNA and mRNA expression profiling on renal biopsy sections of clinically stable and progressive CKD patients and identified specific molecules associated with disease progression^14^. However, to our knowledge, all the previous studies on miRNAs urinary expression were either performed on diabetic patients with a clinical diagnosis of DN or solely on diabetic patients with biopsy proven DN. Thus, the tissue expression of miRNAs in diabetic kidney samples with different histological damage, such as non-diabetic lesions in diabetic patients, has not yet been investigated.

The ubiquitination pathway is one among the several mechanisms modulated by the diabetic milieu that can favor the progression of renal damage in diabetic patients. Lysine 63-linked ubiquitylation in particular, is a post-translational modification associated to the non-proteolytic regulation of numerous signaling pathways and is dependent on the specific activity of UBE2v1, also called UEV1a, a co-factor of UBC13, the E2 ubiquitin-conjugating enzyme involved into the formation of lysine63(K63)-linked ubiquitin chains^15^. Notably, we previously demonstrated that urines of patients with DN are enriched in free ubiquitin^16^ and that an accumulation of K63 ubiquitinated proteins at the tubular level is involved in epithelial-to-mesenchymal transition (EMT), driving the progression of tubular-interstitial fibrosis and tubular damage in DN patients^17,18^.

The aim of our study was to explore the pattern of miRNA expression in kidneys and urines collected from diabetic patients with different types of histologically-defined renal damage in order to identify specific candidate biomarkers of DN in diabetic patients.

## Results

### The histological damage in diabetic kidneys reflects distinct miRNA profiles

**Figure 1:**
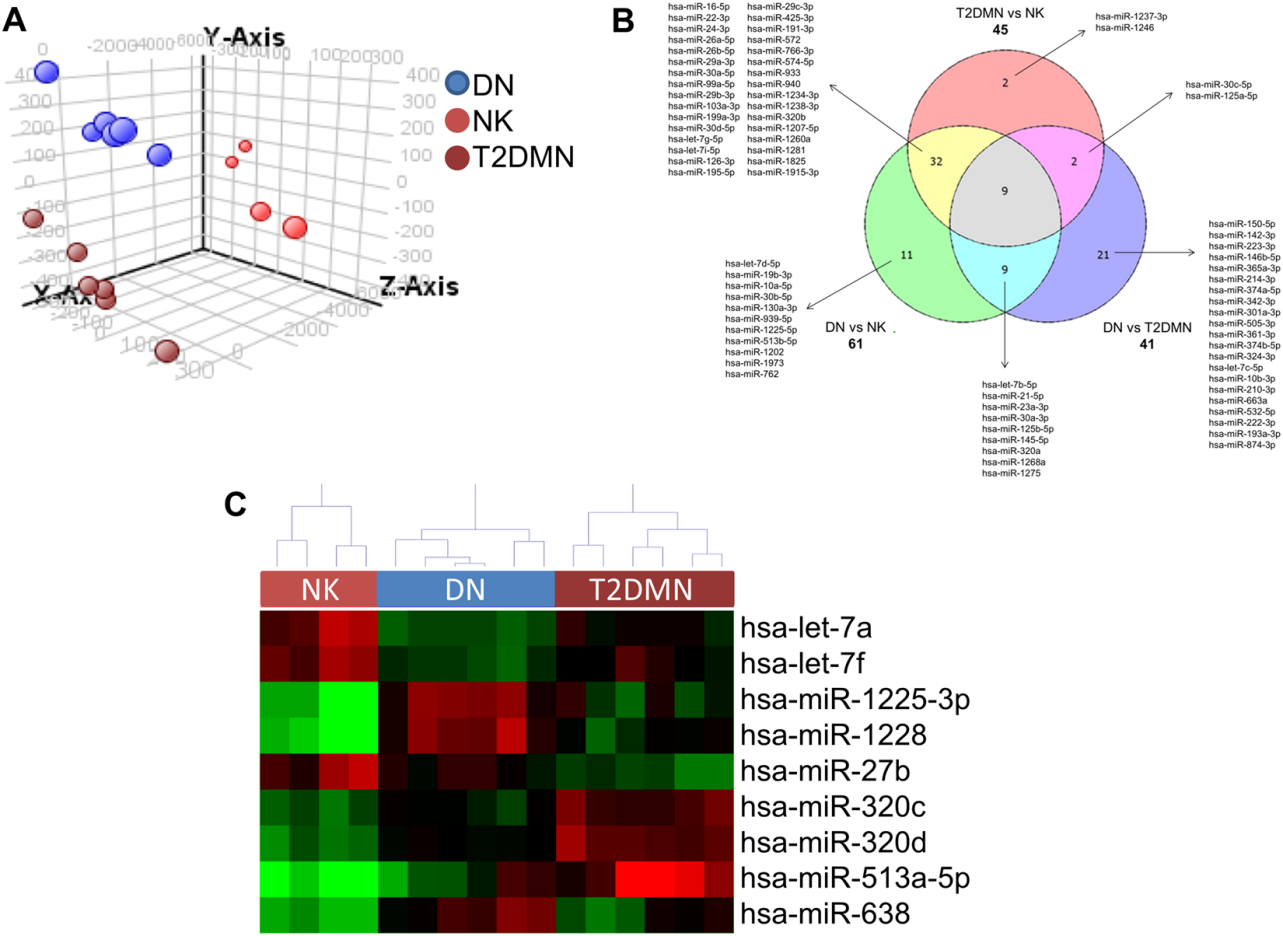
Analysis of miRNAs differentially expressed in kidney biopsies from patients with diabetic nephropathy (DN), patients with diabetes and membranous nephropathy (T2D-MN), or patients with normal kidney (NK). (**A**) Principal component analysis (PCA) built on all expressed miRNAs among the three patients’ groups. (**B**) Venn diagram built on miRNAs differentially expressed with FC ≥ 1.5 when comparing the different groups of patients. The list of the specific miRNAs included in each comparison and common among the different evaluations is reported. (**C**) Hierarchical clustering built on the 9 miRNAs common to all three comparisons described in panel B (in grey in the Venn diagram). Expression levels of these miRNAs were completely different among the three groups of patients.

In order to evaluate whether diabetic kidney damage is characterized by different miRNA profiles, we performed an array-based miRNome profiling on total RNA extracted from 6 DN, 6 T2D-MN, and 4 NK kidney biopsy tissues. Principal Component Analysis (PCA) revealed complete separation of the patient groups, based on the expression profiles of all miRNAs (Fig. 1A). By applying a fold change = 1.5, FDR ≤ 0.05, we identified 61 miRNAs differentially expressed in DN when compared to NK, 41 miRNAs differentially expressed in DN when compared to T2D-MN and 45 miRNAs differentially expressed in T2D-MN when compared to NK (Fig. 1B and Supplementary Table S1). Venn diagram revealed that 9 miRNAs were differentially expressed among all the three groups of patients, thus suggesting their potential role as markers of specific histological damage. Hierarchical clustering using these 9 miRNAs confirmed different expression profiles among the patient groups (Fig. 1C).

Since our group has previously demonstrated that K63-linked ubiquitylation represents a novel mechanism involved in the progression of tubular damage and fibrosis in DN^17^, among the 9 specific miRNAs differentially expressed in DN patients when compared to T2D-MN and NK, we selected those that specifically target UBE2v1, the E2 enzyme involved in K63-linked ubiquitylation. Using different freely available databases (TargetScan, DIANA MicroT-CDS, miRDB, miRTarBase, DIANA TarBase v7.0) we identified in particular two miRNAs: miR-1228-3p, with UBE2v1 as lightly predicted target in TargetScan and experimentally validated target in miRTarBase^19^, although the specific interaction was not properly demonstrated by authors, and miR-27b-3p, with UBE2v1 as predicted target in TargetScan, DIANA MicroT-CDS and miRDB. Of note, these two miRNAs were found to be downregulated in the microarray dataset in the DN group, and this result was in line with the increased expression of UBE2v1 in DN at the tissue level, as previously demonstrated^17^.

### miR-27b-3p and miR-1228-3p were specifically downregulated in DN kidney compared to T2D-MN

**Figure 2:**
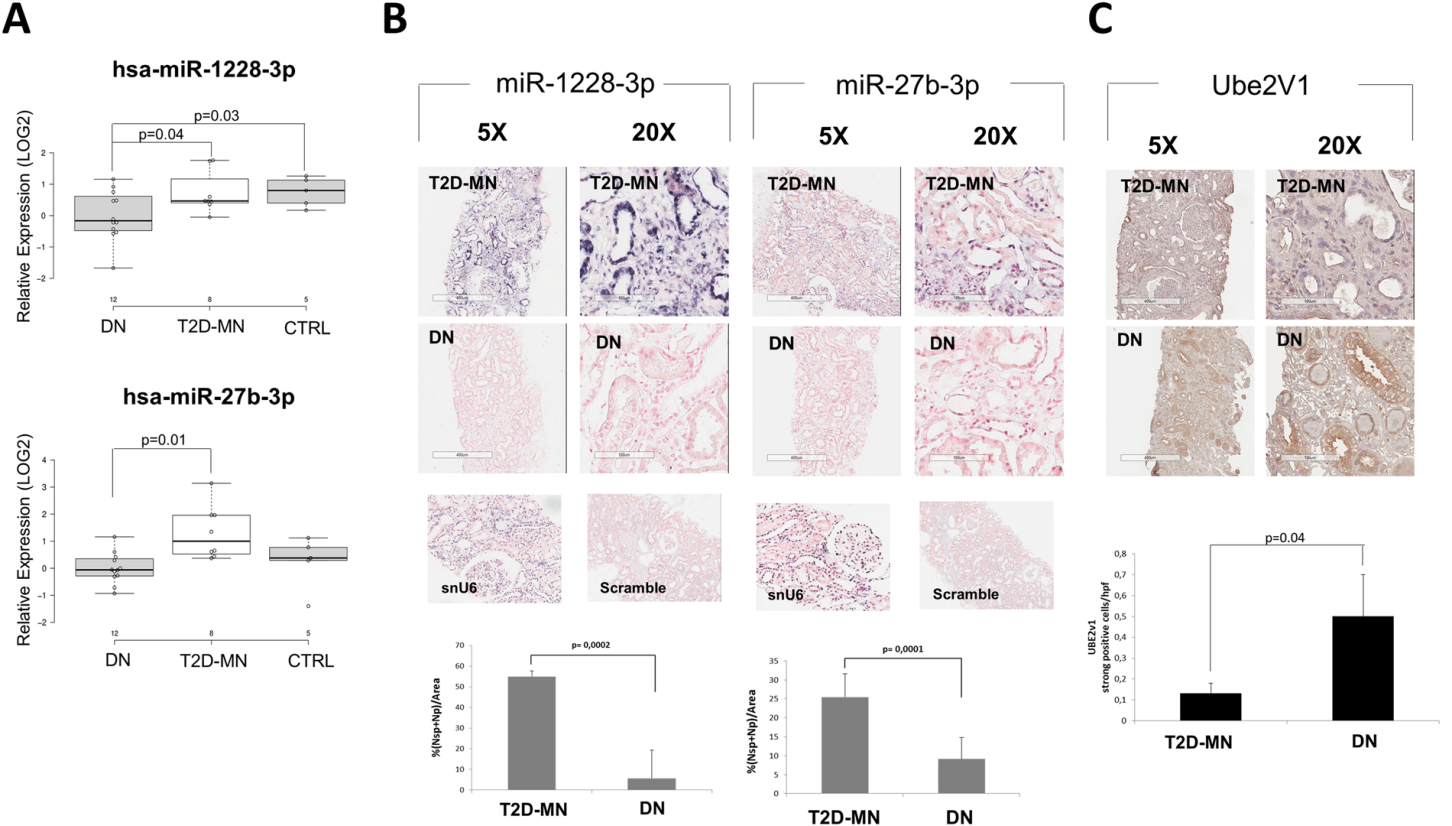
miRNA 27b-3p and miRNA 1228-3p validation on kidney tissues. (**A**) qPCR of miRNA 1228-3p (upper panel) and miRNA 27b-3p (lower panel) expression levels on RNA extracted from kidney biopsies of 5 patients with normal kidney morphology (NK), 12 DN and 8 T2D-MN patients. Center lines show the medians; box limits indicate the 25^th^ and 75^th^ percentiles as determined by R software; whiskers extend 1.5 times the interquartile range from the 25^th^ and 75^th^ percentiles, outliers are represented by dots. (**B**) *In situ* Hybridization of miRNA 1228-3p and 27b-3p expression levels on kidney biopsies of DN and T2D-MN patients. snU6: small nuclear RNA U6 positive control. Scramble: DIG-labeled Scramble-miR probe negative control. ISH quantification was performed as described in methods. The histogram represents the mean ± SD. (**C**) Immunohistochemical analysis on kidney biopsies of patients with T2D and membranous nephropathy (MN) or diabetic nephropathy (DN).

To validate microarray results, we isolated RNA from the kidney biopsies of an independent testing set (validation cohort) composed of 5 patients with normal kidney morphology (NK),12 patients with biopsy-proven DN and 8 diabetic patients with membranous nephropathy (T2D-MN). qRT-PCR for the two selected miRNAs confirmed that miR-27b-3p and hsa-miR-1228-3p were significantly down-regulated in DN kidney tissues vs T2D-MN biopsies, with miR-1228-3p also significantly down-regulated in DN patients compared to NK (Fig. 2A).

Finally, to further confirm the differential expression and analyze the localization of hsa-miR-27b-3p and hsa-miR-1228-3p in DN kidneys compared to T2D-MN kidneys, we performed *in-situ* hybridization. As shown in Fig. 2B, hsa-miR-1228-3p and hsa-miR-27b-3p were significantly reduced in DN biopsies compared to T2D-MN. To further confirm that miR-27b-3p and miR-1228-3p directly affect UBE2v1 expression, we investigated the protein expression of UBE2v1 by immunohistochemistry on the same biopsy samples. Results confirmed that in DN patients the down-regulation of both miRNAs was inversely associated with an increased expression of UBE2v1 protein when compared to T2D-MN (Fig. 2C).

### miRNA analysis of urinary samples confirmed that miR-27b-3p and miR-1228-3p were specifically down-regulated in DN patients

**Figure 3:**
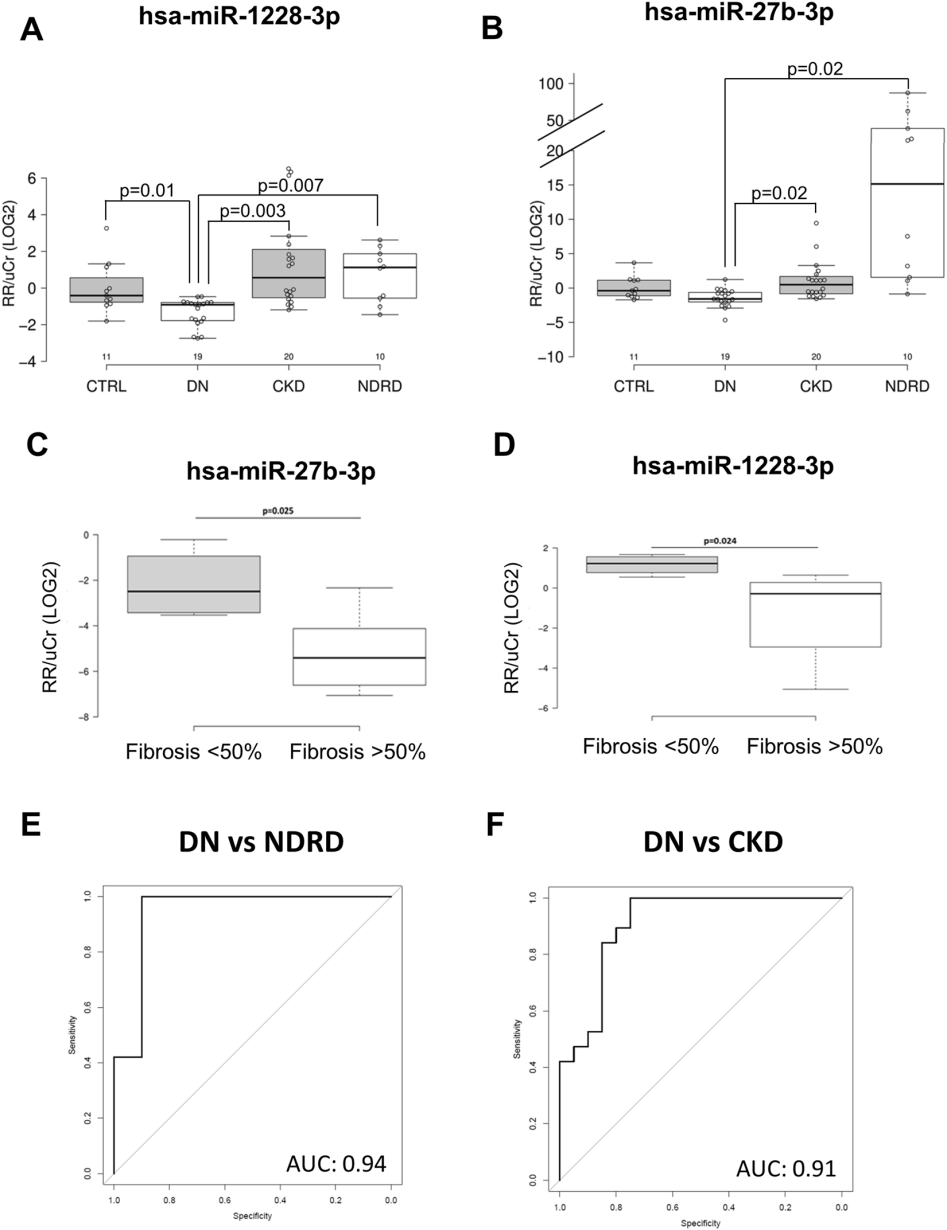
miRNA 27b-3p and 1228-3p urinary expression levels. (**A**,**B**) qPCR of miRNA 27b-3p (A) and 1228-3p (B) expression levels on RNA extracted from cell free urines of 11 healthy subjects (CTRL), 20 CKD, 10 NDRD and 19 DN patients. Center lines show the medians; box limits indicate the 25^th^ and 75^th^ percentiles as determined by R software; whiskers extend 1.5 times the interquartile range from the 25^th^ and 75^th^ percentiles, outliers are represented by dots. (**C**,**D**) Pearson correlation between miRNA 27b-3p (**C**) and 1228-3p (**D**) urinary expression levels and kidney fibrosis quantified as described in methods. (**E**,**F**) ROC curve describing the sensitivity and specificity of miRNA 27b-3p and 1228-3p combined urinary expression levels to discriminate DN vs NDRD (**E**) and DN vs CKD (**F**).

We then analyzed the urinary miRNA expression in a different validation cohort of patients with normal and compromised renal function using Real-Time PCR. For this purpose, we used RNA isolated from cell-free urine samples of healthy volunteers (n = 11), diabetic patients with biopsy-proven DN (n = 19), diabetic patients with biopsy-proven NDRD (n = 10), non-diabetic patients with CKD (n = 20). Interestingly, both miR-27b-3p and miR-1228-3p were significantly downregulated in DN urines compared to all the indicated groups (Fig. 3A,B).

### miR-27b-3p and miR-1228-3p expression was directly correlated to the degree of fibrosis in DN and discriminated the extent of histological damage

Since we previously observed that K63-linked ubiquitylation is involved in epithelial to mesenchymal transition, thus driving the progression of renal fibrosis in DN patients, we analyzed the correlation between urinary levels of miRNA and the degree of tissue fibrosis by Masson’s trichrome staining. To this purpose, all patients enrolled in the study were classified in two groups according to the degree of renal fibrosis. In particular, patients showing more than 50% fibrosis were classified in one group, while those showing less than 50% of renal fibrosis were classified into the other group. Interestingly, urinary levels of both miRNAs were able to discriminate between the two different degrees of renal fibrosis (Fig. 3C,D).

Finally, the AUC of the ROC curve derived from the logistic regression model (Supplementary Table S2) including the two miRNAs to predict the diagnosis of DN versus NDRD was 0.94 (95% CI: 0.8264-1) with a specificity of 0.90 and a sensitivity of 1.0 (Fig. 3E) while the AUC of the ROC curve derived from the logistic regression model (Supplementary Table S2) including the two miRNAs to predict the diagnosis of DN versus CKD was 0.91 (95% CI: 0.8141–1) with a specificity of 0.75 and a sensitivity of 1.0 (Fig. 3F).

### Hyperglycemia affects miR-1228–3p expression by DNA methylation

**Figure 4:**
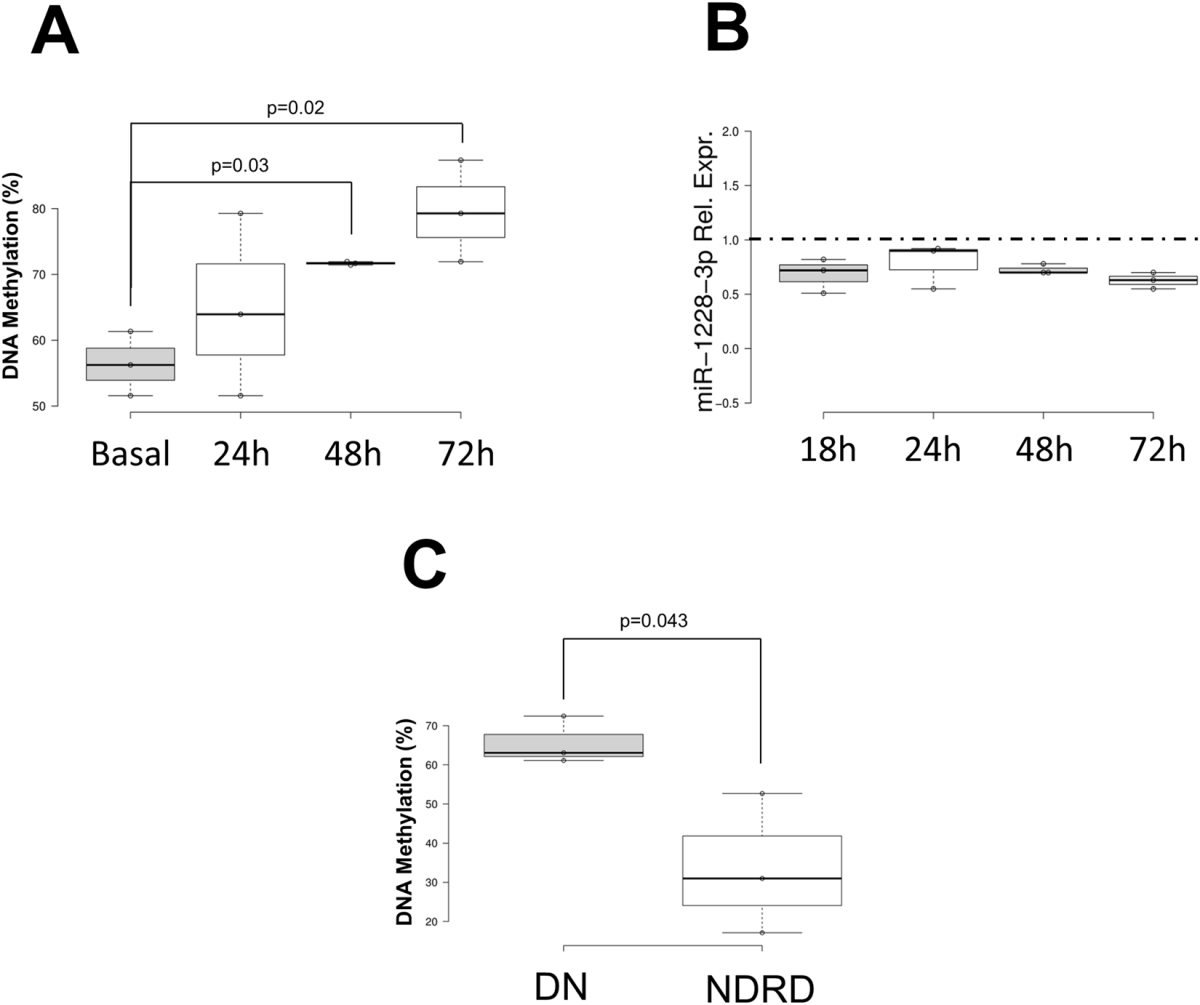
Effects of hyperglycemia on miR-1228-3p DNA methylation *in vitro* and *in vivo*. (**A**) DNA methylation levels of miR-1228 in HK2 tubular cells under hyperglycemic conditions at different time points. (**B**) Real-Time PCR of miR-1228-3p expression levels in HK2 cells under hyperglycemic conditions at different time points. (**C**) miR-1228 DNA methylation levels on renal biopsies from 3 DN and 3 NDRD patients.

In order to understand the potential mechanisms responsible for miRNAs down-regulation, we analyzed the methylation levels in the promoter regions of miR-1228 and miR-27b genes, both in renal tubular cells under hyperglycemic conditions (HK2) and in renal biopsies of patients with DN. We performed a bioinformatic analysis to identify CpG islands (regions with GC content of 50% or greater and length greater than 200 bp) and CpG sites within and upstream of the promoter regions (−1000 to +200 bp from transcription site) of these miRNAs and found a predicted CpG island overlapping the miR-1228 gene promoter (chr12:57587579-57587898, GRCh37.p13 assembly) and several CpG sites in miR-27b gene promoter. When HK2 tubular cells were exposed to high glucose, the DNA methylation levels of miR-1228-3p increased progressively over time (Fig. 4A), and this was associated with a trend in the decrease of miR-1228-3p levels (Fig. 4B). When measuring the miR-1228-3p DNA methylation levels on renal biopsies from 3 DN and 3 NDRD patients, we confirmed that, miR-1228-3p promoter was hyper-methylated in DN compared to NDRD (Fig. 4C). In contrast, we did not find differences in DNA methylation in the miR-27b promoter, neither in cells nor in tissues (data not shown), limited to the genomic region analyzed.

## Discussion

In the present paper we demonstrated that: a) different miRNAs expression profiles reflect different types of renal damage in diabetic patients; b) miR-27b-3p and miR-1228-3p, which are predicted to target the UBE2v1 gene that promotes the accumulation of K63-linked ubiquitinated proteins, are specifically downregulated in kidney biopsies of DN patients; c) miR-27b-3p and miR-1228-3p downregulation was still present in urines of DN patients and correlated with the degree of kidney fibrosis; d) miR-27b-3p and miR-1228-3p urinary levels were able to discriminate DN from NDRD and DN from CKD; and e) miR-1228-3p was regulated by altered glucose-dependent methylation at specific CpG sites in DN kidneys.

The differential diagnosis between DN and NDRD in patients with diabetes and albuminuria has been underestimated over the years^9^. Recent studies have highlighted the importance kidney biopsy in diabetic patients with compromised renal function to detect non-DN histological lesions in diabetic patients with albuminuria^5^. Ample evidence from the literature suggests that novel potential biomarkers can help in the diagnosis of DN^10,20–22^. In the majority of cases, however, samples such as urine and other biological fluids were collected from patients classified as DN solely on the basis of their clinical features. This could represent a major bias as it is highly plausible that a portion of diabetic patients with NDRD might have been erroneously included in the DN cohort^4,23^. The aim of our study was therefore to focus on a well-defined cohort of diabetic patients with complete clinical and histological information and to investigate whether the heterogeneity of the histological lesions was associated with a different expression of miRNAs both in the kidney and urine.

The analysis of small molecules such as miRNAs, known to be actively and passively secreted in different biological fluids, has emerged as an additional tool for diagnosis^20^. Tissue-specific microRNA expression patterns have been recently described in four types of kidney disease including DN^24,25^. In a recent systematic review and computational analysis, Assmann *et al*. also identified differentially expressed miRNAs in patients with diabetic kidney disease^26^, results however are still inconclusive since they lack information regarding the association between miRNA signatures in diabetic kidneys with different histological phenotypes. In this setting, we identified two miRNAs, at both urinary and tissue level, able to discriminate DN patients from diabetics with different types of renal damage and non-diabetic patients with CKD; of note, these miRNAs also correlate with the degree of renal fibrosis. Several miRNAs have been described as involved into the progression of kidney fibrosis in DN^27^ as for example miR-30c^28^, miR-27a^29^, miR-130b^30^ and miR-192^25^. In addition, we have previously identified a novel molecular mechanism responsible for miR-21-mediated aberrant ECM turnover in DN, showing that induction of this miRNA is at least partially responsible for the downregulation of tissue inhibitor of metalloproteinase-3 (TIMP3)^31^. In this study, we describe for the first time the involvement of miR-27b-3p and miR-1228-3p in the regulation of kidney fibrosis in DN, possibly through the modulation of K63-linked protein ubiquitylation. However, since miRNAs can regulate several targets simultaneously, different mechanisms other than K63- linked protein ubiquitylation, could be affected by de-regulation of miR-27b-3p and miR-1228-3p in DN patients. Several studies from the literature support the involvement of miR-27b-3p and miR-1228-3p in the progression of fibrosis: Akhtar *et al*. found that MMP13, a protein responsible for the physiological breakdown of extracellular matrix, is a known target for miR-27b-3p^32^; MMPs seem to act as the pathogenic mediators of kidney fibrosis and the assessment of specific MMPs in urinary samples was proposed as a noninvasive tool for the detection of kidney fibrosis^33^. Down-regulation of miR-27b-3p was also reported to have a role in the pathophysiology of heart failure, involved in pathways related to disease progression, including heart fibrosis^34^. The modulation of miR-27b-3p expression has been also described in the context of diabetes; in a recent meta-analysis, Liang *et al*. described miR-27b as a potential circulating biomarker of type 2 diabetes^35^. Finally, miR-27b-3p was described as involved in insulin resistance^36^ as well as a novel biomarker of diabetic retinopathy in type 1 diabetes^37^.

On the other hand, miR-1228 plays an important role in the regulation of cell proliferation and apoptosis in cancer cells^38,39^. Jia *et al*. also described the involvement of miR-1228 in the regulation of fibrosis and demonstrated that restoration of the expression levels of this miRNA suppresses epithelial to mesenchymal transition in gastric cancer^40^. Moreover, in the context of diabetes, Satake *et al*. identified, among others, miRNA-1228-3p as negatively correlated with HbA1c in patients with type 1 diabetes mellitus^41^, thus underlying that chronic hyperglycemia can affect the expression levels of this miRNA. One possible mechanism for the regulation of gene expression is represented by epigenetic changes, such as DNA methylation. In DNA samples collected from diabetic patients, Maghbooli *et al*. reported that global methylation levels were significantly higher in patients with albuminuria compared to those with normoalbuminuria^42^. Recently, employing a genome-wide cytosine methylation profiling of tubule epithelial cells obtained from CKD and control kidneys, Ko *et al*. identified different cytosine methylation patterns within the enhancer regions of important pro-fibrotic genes such as collagen, suggesting that during diabetes methylation is indeed affected in renal cells also^43^. In the present paper, we finally report, both *in vitro*, in HK2 tubular cells, and *in vivo*, on DNA extracted from kidney biopsies, the effect of hyperglycemia on the methylation of specific CpG sites corresponding to important regulatory regions for miRNA-1228-3p transcription. Preliminary results suggest that down-regulation of miR-1228-3p at tubular level in DN patients is associated to hyper-methylation in the promoter region of this miRNA. Indeed, further investigation is necessary to analyze other CpG sites in a greater number of patients to definitively confirm the role of glucose-induced DNA hyper-methylation in the regulation of miR-1228-3p expression.

Our study has a major limitation related to the small sample size, but we need to point out that, unlike in other studies, all the patients enrolled in this study were extensively defined histologically. Indeed, the validation of these miRNAs as biomarker candidates of diagnosis and prognosis will need to be further confirmed in a larger prospective study, possibly including diabetic patients with normal renal function that are monitored over time. This will be helpful to define the miRNA expression cut-off that predicts the onset of renal damage. Moreover, the described model could be implemented with additional clinical data associated to DN occurrence to allow for a non-invasive diagnosis of DKD.

In conclusion, our data describe two novel miRNAs that are able to discriminate different histological lesions in diabetic and CKD patients. The down-regulation of miR-27b-3p and miR-1228-3p, initially detected at tissue level and maintained in urine, renders miR-27b-3p and miR-1228-3p two possible non-invasive urinary candidate biomarkers for DN diagnosis and prognosis.

## Methods

### Patient characteristics

The present study was approved by the Independent Ethics Committee of the Azienda Ospedaliero Universitaria Policlinico Consorziale di Bari; all patients gave written informed consent for the use of this material for research purposes and the study was conducted in accordance with the Helsinki Declaration (Prot. N.4104/2013).

Biopsy specimens were evaluated through light, immunofluorescence and electron microscopy independently by two renal pathologists, blinded to patients’ features. Patients with urinary abnormalities (hematuria and/or albuminuria and/or proteinuria) but with a normal kidney morphology were used as control (NK). Patients with DN showed generalized thickening of glomerular basement membranes (GBMs) and diffuse mesangial matrix expansion or Kimmelstiel-Wilson nodular appearance or, in most cases, a combination of both; arteriolar hyalinosis, commonly affecting the glomerular hilum; occasionally fibrin necrosis of the afferent arterioles, fibrin caps and mild mesangial hypercellularity; glomerular non-specific staining for IgG and C3. Patients with membranous nephropathy (MN) showed a global thickening of the GBMs with sub-epithelial immune deposits. Patients with focal segmental glomerulosclerosis (FSGS) showed the classical tip lesions as well as floccular adherence or collapsing appearance.

#### Discovery cohort

A total of 16 RNA samples were analyzed using microarray technology; 4 were from kidney biopsies of patients with NK; 6 were from kidney biopsies of type 2 diabetic patients with DN; 6 were from kidney biopsies of diabetic patients with MN (T2D-MN).

#### Validation cohort

For miRNA validation in kidney tissue, a total of 20 RNA samples were analyzed using Real-Time PCR; 12 were from diabetic patients with DN; 8 were from diabetic patients with NDRD (T2D-MN, T2D-FSGS).

For miRNA validation in cell-free urine, a total of 60 RNA samples were analyzed using Real-Time PCR: 11 were from healthy volunteers; 19 were from diabetic patients with DN; 10 were from diabetic patients with NDRD and 20 were from non-diabetic patients with CKD. The clinical features of the discovery and validation cohorts are detailed in Table 1.

**Table 1:** Main demographic and clinical characteristics of patients enrolled in the study belonging to the discovery cohort (upper panel) and to the validation cohort (lower panel).

Clinical Characteristics	DN (n = 6)	T2MN (n = 6)	NK (n = 4)	
***Discovery Cohort***				
Sex (M/F)	4/2	6/0	3/1	
Age (years)	59 ± 16	63 ± 16	38 ± 14	
Duration of Diabetes (years)	15 ± 7	13 ± 4	0	
Systolic Pressure (mmHg)	135 ± 20	140 ± 15	120 ± 10	
Diastolic Pressure (mmHg)	80 ± 10	74 ± 9	75 ± 6	
BMI (kg/m^2^)	33 ± 4	26 ± 4	26 ± 4	
eGFR CKD-EPI(mL/min/1.73 m^2^)	33 ± 20	78 ± 39	102 ± 30	
Serum Creatinine (mg/dl)	2,9 ± 2,4	1,43 ± 1,26	0,83 ± 0,14	
Proteinuria (mg/24 h)	8982 ± 6267	5080 ± 3986	460 ± 250	
**Clinical Characteristics**	**DN (n** = **19)**	**Diabetic NDRDs (n** = **10)**	**CKD (n** = **20)**	**CTRLs (n** = **11)**
***Validation Cohort***				
Sex (M/F)	11/8	6/4	15/5	5/6
Age (years)	64 ± 10	59 ± 17	56 ± 18	43 ± 12
Duration of Diabetes (years)	17 ± 11	14 ± 15	0	0
Systolic Pressure (mmHg)	140 ± 20	129 ± 16	141 ± 29	115 ± 8
Diastolic Pressure (mmHg)	80 ± 15	73 ± 15	81 ± 11	72 ± 4
BMI (kg/m^2^)	30 ± 9	28 ± 5	27 ± 3	24 ± 3
eGFR CKD-EPI(mL/min/1.73 m^2^)	38 ± 29	89 ± 30	83 ± 35	101 ± 10
Serum Creatinine (mg/dl)	2,62 ± 1,54	0,99 ± 0,57	1,16 ± 0,65	0,87 ± 0,09
Proteinuria (mg/24 h)	3856 ± 3225	4440 ± 5154	5766 ± 4755	110 ± 21
ACR (mg/g)	1350 ± 1072	1784 ± 1611	1789 ± 1388	21 ± 3
Diabetic Retinopathy (%)	63	33	0	0
Oral Hypoglycemic Agents (%)	40	71	0	0
Insulin (%)	65	0	0	0
Hypoglycemic Diet only (%)	0	29	0	0
ACEi and/or Sartans (%)	100	86	68	0
Total Cholesterol (mg/dl)	160 ± 55	188 ± 57	236 ± 65	132 ± 21
HDL Cholesterol (mg/dl)	34 ± 11	36 ± 6	63 ± 24	52 ± 15
LDL Cholesterol (mg/dl)	86 ± 34	88 ± 34	139 ± 54	88 ± 31
Triglycerides (mg/dl)	205 ± 112	197 ± 70	160 ± 57	116 ± 25
Glycated Hemoglobin (%)	7,2 ± 1,5	5,7 ± 1,6	5,1 ± 0,9	5,1 ± 0,1

### RNA Isolation

#### Kidney biopsies

Total RNA, including miRNAs, was extracted from formalin-fixed, paraffin-embedded (FFPE) sections with a 5 μm thickness using the miRNeasy FFPE Kit (Qiagen, Germantown, MD) according to the manufacturer’s instructions. To ensure the complete elimination of genomic DNA, a DNAse digestion step was performed during extraction. Following purification, RNA concentration and purity were assessed using NanoDrop ND-1000 Spectrophotometer (ThermoFisher Scientific, Waltham, MA, USA).

#### Urine samples

RNA from urine was isolated using the miRNeasy Serum/Plasma kit (Qiagen) according to the manufacturer’s instructions. In brief, 1 mL of QIAzol reagent was added to 200 µl of cell-free urine. Sample was mixed in a tube followed by the addition of 200 µl of chloroform. After vigorous mixing the sample was centrifuged at 12,000 × g for 15 minutes at 4 °C. The upper aqueous phase was carefully transferred to a new collection tube and loaded into the QIAcube robotic workstation for automated RNA extraction. The exogenous Cel-miR-39 spike-in (Qiagen, Cat No.: 219610) was added in equal amounts to all samples prior to RNA isolation to monitor the efficiency of RNA extraction. Total RNA was eluted in 15 μL of RNase-free water and stored at −80 °C until further use.

### Microarray hybridization

RNA samples (100 ng) were processed for microarray hybridization using the miRNA complete labeling and hyb kit (Agilent Technologies, Santa Clara, CA, USA) according to manufacturer’s instructions (protocol V2.4). The labeled RNA was hybridized onto the Agilent Human miRNA Microarray, Release 14.0, 8 × 15 K slides; these slides are based on Sanger miRBase release 14.0 and covered 884 distinct Human miRNAs. The miRNA expression data has been deposited on NCBIs Gene Expression Omnibus (GEO, http://www.ncbi.nlm.nih.gov/geo/) and is accessible through the GEO series accession number GSE51674.

### MiRNA target analysis

Validated and predicted interactions between the enzyme UBE2v1 and our miRNA dataset were identified using several computational algorithms: TargetScan^44^, DIANA MicroT-CDS^45^, miRDB^46^, miRTarBase^47^ and DIANA TarBase v7.0^48^.

### Real-Time PCR validation and data analysis

For RNA isolated from FFPE kidney biopsies, the total RNA input for retro-transcription was 25 ng; for urine samples, 2 μL of total RNA were used. RNA was retro-transcribed using the miScript II RT Kit and HiSpec Buffer (Qiagen). cDNA was then diluted 50x and assayed in 10 μL PCR reactions; each miRNA was assayed in duplicate by qPCR using the miScript SYBR Green PCR Kit and the following primers: hsa-miR-27b-3p miScript Primer Assay - cat. no. MS00031668; hsa-miR-1228-3p miScript Primer Assay - cat. no. MS00042385; Hs_SNORD61_11 miScript Primer Assay cat. no. MS00033705.

Amplification was performed in a LightCycler® 96 Real-Time PCR System (Roche Diagnostics, Indianapolis, IN) in 96-well plates. The amplification curves were analyzed using the Roche LC software, both for melting curve analysis and for determination of Cq by the 2nd derivative method^49^. All assays were inspected for distinct melting curves and the Tm was checked to be within known specifications for the assay. For the analysis of miRNAs in renal biopsies, data was normalized to the small nucleolar RNA SNORD61. In urinary samples, miRNA expression level was normalized to the cel-miR-39 (Caenorhabditis elegans miR-39) spike-in control and calculated through the equation: ΔCq = Cq_target_ − Cq_Cel39_. Finally we divided the relative levels of miRNA expression (2^−dCt^) by urinary creatinine for each patient to account for urinary dilution.

### *In-Situ* hybridization on FFPE kidney

*In-situ* hybridization was performed on 6 μm thick paraffin sections according to the miRCURY LNA™ microRNA ISH protocol (EXIQON). Digestion was carried for 15 minutes at 37 °C using 10 μg/mL Proteinase K; sections were then incubated with the double DIG-labeled miRCURY LNA™ microRNA 1228-3p probe (EXIQON, cat. no. 612530-360) and microRNA 27b-3p probe (EXIQON, cat. no. 611167-360) at a concentration of 30 nM each. Hybridization was performed at 60 °C for 1 h. For signal detection, tissues were incubated with the sheep anti-DIG-AP antibody (ROCHE) at a 1:250 concentration. Slides were scanned at a 20x magnification using the ScanScope system (Aperio Technologies). Quantification was performed using the Positive Pixel Count v9_v10.0.0.1805 algorithm (Aperio Technologies) to measure the number of positive cells (Np) and strong positive cells (Nsp) referred to the area (%Nsp + Np/Area).

### UBE2v1 immunohistochemistry on kidney biopsies

Immunohistochemistry was performed as previously reported (17). Briefly, paraffin-embedded tissue underwent re-hydration and antigenic retrieval and were incubated with H_2_O_2_ (3%) and then with Triton (0.25%). After blocking with Protein block (Dako, Glostrup, Denmark), sections were incubated with anti-UBE2v1 specific antibody (Abcam, Cambridge, UK) and the immune-complexes were detected by the Peroxidase/DAB Dako Real EnVision Detection System (Dako). Digital images were acquired using Aperio ScanScope CS2 device (Aperio Technologies, Vista, CA) at 20X magnification and analyzed by ImageScope V12.1.0.5029 (Aperio). Staining was quantified using the Positive Pixel Count v9_v10.0.0.1805 algorithm (Aperio Technologies) to measure the number of positive cells. For each region (hpf) the number of cells with strong (3+) signal identified by the algorithm were considered.

### Purification of DNA and RNA from cells and tissues

DNA and RNA were purified simultaneously from both tubular cells and flash frozen renal biopsies of 3 DN and 3 NDRD patients. The HK-2 (human kidney 2) proximal tubular cell line was purchased from the American Type Culture Collection (ATCC). HK-2 cells were cultured in DMEM supplemented with 10% FBS and different glucose concentrations (5.5 mM and 30 mM). The absence of mycoplasma was assessed using the MycoAlert Mycoplasma Detection Kit (Lonza) according to manufacturer’s instructions. Nucleic acids were purified using the AllPrep DNA/RNA/miRNA Universal Kit (QIAGEN) according to manufacturer’s instruction. DNA and RNA were quantified using Nanodrop ND-1000 Spectrophotometer and stored at −80 °C.

### Quantitative methylation-specific PCR assay (qMSP)

Bisulfite converted DNA was used to perform the qMSP by Methylamp MS-qPCR Fast Kit (Epigentek Group) according the manufacturer instructions. For each reaction 20 ng of bisulfite-treated DNA was used as template. Primers for the genes of interest were designed using MethPrimer (http://www.urogene.org/methprimer/index.html) (miRNA-27b-3p_FOR: TAGTGTGTGTAGATAGTACGGGG; miRNA-27b-3p_REV: CTACAATAAACGCGACCACG; miRNA-1228-3p_FOR: ATAGAGAAAATTTTGGTTTGACGA; miRNA-1228-3p_REV: CTAATTACAACGACTAAAATTCCCG). ACTB (β-actin) gene was used as a reference gene. No-template controls were included in each run as negative controls. An EpiTect Control DNA, a 100% methylated DNA (Qiagen), was used as a positive control for all genes studied. The PMR (percentage of methylated reference) (i.e. degree of methylation) was used to define the percentage of fully methylated molecules at a specific locus and was calculated as reported previously^50^. Briefly, the PMR value was calculated by dividing the gene/ACTB ratio in a sample by the gene/ACTB ratio in SssI-treated leucocyte DNA (Qiagen) and multiplied by 100. Parallel PCRs were carried out for the genes of interest and reference. PMR values were detected using the comparative CT method. The relationship between the percentages of methylated DNA molecules and CT is described as PMR = 2^−DDCT^ × 100%.

### Masson’s trichrome staining

Masson’s trichrome staining on kidney tissue was performed as previously described^51^. Briefly, 2 μm-thick paraffin-embedded renal biopsy sections were dewaxed and incubated with Bouin solution overnight. Slides were stained in Weigert’s iron hematoxylin solution then stained with scarlet-acid fuchsine solution. After differentiation in acetic acid solution, slides were immersed in green light solution 2% and mounted with Eukitt. Digital slides were then acquired by Aperio ScanScope CS2 device (Aperio Technologies, Vista, CA). Green stained area was quantified using Adobe Photoshop software and expressed as positive pixel/total pixel by two independent observers blinded to the origin of the slides. The final quantification was the mean of the two measurements.

### Statistical analysis

Statistical analysis of microarray data was performed using GeneSpring GX 12.5 software (Agilent Technologies): after quantile normalization and quality control, statistical significance of the differentially expressed miRNAs was assessed by unpaired t-test with Benjamini-Hochberg multiple testing correction using a p-value cut-off of 0.05 and a fold-change of 1.5.

ROC curve analysis, based on predicted probability values from logistic regression models built from the validation cohort, was used to evaluate the diagnostic performance of the two miRNAs for the detection of diabetic nephropathy. Internal validation was performed by bootstrapping using 1000 samples. Statistical analyses were carried out using R version 3.4.3. (R Core Team (2017). R: A language and environment for statistical computing. R Foundation for Statistical Computing, Vienna, Austria. https://www.R-project.org/).

Data are presented as mean ± standard deviation (SD) and compared by unpaired t-test. P < 0.05 was considered statistically significant.

## Supplementary information

Dataset 1

## Data Availability

The dataset generated during the current study is available in the GEO repository, series accession number GSE51674.
