# Supplementary material for: Urinary miRNA-27b-3p and miRNA-1228-3p correlate with the progression of Kidney Fibrosis in Diabetic Nephropathy
Source: Sci Rep. 2019 Aug 6;9:11357. doi: 10.1038/s41598-019-47778-1 (PMC6684817; doi:10.1038/s41598-019-47778-1)
Supplement: Supplementary file 1 — Dataset 1 [file 41598_2019_47778_MOESM1_ESM.pdf]

Urinary miRNA-27b-3p and miRNA-1228-3p correlate with the progression of Kidney Fibrosis in Diabetic Nephropathy

Francesca Conserva[§1], Mariagrazia Barozzino[§1], Francesco Pesce[1], Chiara Divella[1], Annarita Oranger[1], Massimo Papale[1], Fabio Sallustio[3], Simona Simone[1], Luigi Laviola[2], Francesco Giorgino[2], Anna Gallone[3], Paola Pontrelli[°*1] and Loreto Gesualdo[°1]

[1] Department of Emergency and Organ Transplantation - Nephrology Unit, University of Bari Aldo Moro, Bari, Italy

[2] Department of Emergency and Organ Transplantation – Section of Internal Medicine, Endocrinology, Andrology and Metabolic Disease, University of Bari Aldo Moro, Bari, Italy

[3] Department of Basic Medical Sciences, Neurosciences and Sense Organs, University of Bari Aldo Moro, Bari, Italy

[§]These two authors equally contributed as first authors

° These two authors equally contributed as senior authors

[*]**Corresponding author:** Paola Pontrelli

Department of Emergency and Organ Transplantation - Nephrology Unit

University of Bari Aldo Moro, Piazza Giulio Cesare, 11, 70124 Bari, ITALY

Telephone: +39 080 5478868; Fax: +39 080 5478 143

E-mail: *paola.pontrelli@uniba.it*

Supplementary Table 1: Fold change of deregulated miRNAs in diabetic kidneys and controls

miRBase Accession No	miRBase Rel. 14	miRBase Rel. 21	DN vs NK	DN vs T2DMN	T2DMN vs NK
MIMAT0000062	hsa-let-7a	hsa-let-7a-5p	3.91	1.73	2.25
MIMAT0000063	hsa-let-7b	hsa-let-7b-5p	2.55	2.19	
MIMAT0000064	hsa-let-7c	hsa-let-7c-5p		1.73	
MIMAT0000065	hsa-let-7d	hsa-let-7d-5p	1.92		
MIMAT0000067	hsa-let-7f	hsa-let-7f-5p	3.22	1.64	1.97
MIMAT0000069	hsa-miR-16	hsa-miR-16-5p	4.75		3.10
MIMAT0000074	hsa-miR-19b	hsa-miR-19b-3p	2.46		
MIMAT0000076	hsa-miR-21	hsa-miR-21-5p	11.25	3.44	
MIMAT0000077	hsa-miR-22	hsa-miR-22-3p	1.92		2.41
MIMAT0000078	hsa-miR-23a	hsa-miR-23a-3p	3.06	1.73	
MIMAT0000080	hsa-miR-24	hsa-miR-24-3p	2.11		2.51
MIMAT0000082	hsa-miR-26a	hsa-miR-26a-5p	2.78		3.36
MIMAT0000083	hsa-miR-26b	hsa-miR-26b-5p	2.50		2.25
MIMAT0000086	hsa-miR-29a	hsa-miR-29a-3p	4.54		4.44
MIMAT0000087	hsa-miR-30a	hsa-miR-30a-5p	1.94		2.13
MIMAT0000088	hsa-miR-30a*	hsa-miR-30a-3p	-1.75	-1.50	
MIMAT0000097	hsa-miR-99a	hsa-miR-99a-5p	2.51		2.31
MIMAT0000100	hsa-miR-29b	hsa-miR-29b-3p	2.85		2.97
MIMAT0000101	hsa-miR-103	hsa-miR-103a-3p	2.12		2.23
MIMAT0000232	hsa-miR-199a-3p	hsa-miR-199a-3p	4.40		3.16
MIMAT0000244	hsa-miR-30c	hsa-miR-30c-5p		2.10	
MIMAT0000244	hsa-miR-30c	hsa-miR-30c-5p			-2.59
MIMAT0000245	hsa-miR-30d	hsa-miR-30d-5p	-1.83		-1.71
MIMAT0000253	hsa-miR-10a	hsa-miR-10a-5p	1.82		
MIMAT0000267	hsa-miR-210	hsa-miR-210-3p		-2.30	
MIMAT0000271	hsa-miR-214	hsa-miR-214-3p		3.89	
MIMAT0000279	hsa-miR-222	hsa-miR-222-3p		-2.65	
MIMAT0000280	hsa-miR-223	hsa-miR-223-3p		6.58	
MIMAT0000414	hsa-let-7g	hsa-let-7g-5p	3.93		3.11
MIMAT0000415	hsa-let-7i	hsa-let-7i-5p	3.87		3.32
MIMAT0000419	hsa-miR-27b	hsa-miR-27b-3p	1.87	-1.89	3.54
MIMAT0000420	hsa-miR-30b	hsa-miR-30b-5p	1.71		
MIMAT0000423	hsa-miR-125b	hsa-miR-125b-5p	2.45	3.19	
MIMAT0000425	hsa-miR-130a	hsa-miR-130a-3p	3.17		
MIMAT0000434	hsa-miR-142-3p	hsa-miR-142-3p		7.86	
MIMAT0000437	hsa-miR-145	hsa-miR-145-5p	1.94	1.92	
MIMAT0000443	hsa-miR-125a-5p	hsa-miR-125a-5p		2.13	
MIMAT0000443	hsa-miR-125a-5p	hsa-miR-125a-5p			-2.84
MIMAT0000445	hsa-miR-126	hsa-miR-126-3p	2.53		1.93

MIMAT0000451	hsa-miR-150	hsa-miR-150-5p		10.47	
MIMAT0000459	hsa-miR-193a-3p	hsa-miR-193a-3p		-3.35	
MIMAT0000461	hsa-miR-195	hsa-miR-195-5p	3.78		2.40
MIMAT0000510	hsa-miR-320a	hsa-miR-320a	-2.34	-1.86	
MIMAT0000681	hsa-miR-29c	hsa-miR-29c-3p	2.96		4.35
MIMAT0000688	hsa-miR-301a	hsa-miR-301a-3p		2.67	
MIMAT0000710	hsa-miR-365	hsa-miR-365a-3p		3.89	
MIMAT0000727	hsa-miR-374a	hsa-miR-374a-5p		3.47	
MIMAT0000753	hsa-miR-342-3p	hsa-miR-342-3p		2.84	
MIMAT0000762	hsa-miR-324-3p	hsa-miR-324-3p		1.76	
MIMAT0001343	hsa-miR-425*	hsa-miR-425-3p	-17.31		-13.98
MIMAT0001618	hsa-miR-191*	hsa-miR-191-3p	-17.97		-15.94
MIMAT0002809	hsa-miR-146b-5p	hsa-miR-146b-5p		4.23	
MIMAT0002876	hsa-miR-505	hsa-miR-505-3p		2.39	
MIMAT0002877	hsa-miR-513a-5p	hsa-miR-513a-5p	-6.62	4.84	-32.06
MIMAT0002888	hsa-miR-532-5p	hsa-miR-532-5p		-2.46	
MIMAT0003237	hsa-miR-572	hsa-miR-572	-9.36		-3.92
MIMAT0003308	hsa-miR-638	hsa-miR-638	-4.57	-1.92	-2.38
MIMAT0003326	hsa-miR-663	hsa-miR-663a		-2.30	
MIMAT0003888	hsa-miR-766	hsa-miR-766-3p	-11.28		-4.96
MIMAT0004556	hsa-miR-10b*	hsa-miR-10b-3p		-1.89	
MIMAT0004682	hsa-miR-361-3p	hsa-miR-361-3p		2.23	
MIMAT0004795	hsa-miR-574-5p	hsa-miR-574-5p	-5.97		-7.60
MIMAT0004911	hsa-miR-874	hsa-miR-874-3p		-3.56	
MIMAT0004955	hsa-miR-374b	hsa-miR-374b-5p		2.09	
MIMAT0004976	hsa-miR-933	hsa-miR-933	-40.09		-12.49
MIMAT0004982	hsa-miR-939	hsa-miR-939-5p	-6.05		
MIMAT0004983	hsa-miR-940	hsa-miR-940	-10.45		-8.92
MIMAT0005572	hsa-miR-1225-5p	hsa-miR-1225-5p	-3.28		
MIMAT0005573	hsa-miR-1225-3p	hsa-miR-1225-3p	-17.68	-2.29	-7.73
MIMAT0005583	hsa-miR-1228	hsa-miR-1228-3p	-16.98	-2.20	-7.70
MIMAT0005589	hsa-miR-1234	hsa-miR-1234-3p	-18.28		-10.39
MIMAT0005592	hsa-miR-1237	hsa-miR-1237-3p			-7.30
MIMAT0005593	hsa-miR-1238	hsa-miR-1238-3p	-18.64		-14.13
MIMAT0005788	hsa-miR-513b	hsa-miR-513b-5p	-6.01		
MIMAT0005792	hsa-miR-320b	hsa-miR-320b	-1.62		-1.69
MIMAT0005793	hsa-miR-320c	hsa-miR-320c	-1.59	1.80	-2.88
MIMAT0005865	hsa-miR-1202	hsa-miR-1202	-2.86		
MIMAT0005871	hsa-miR-1207-5p	hsa-miR-1207-5p	-2.56		-2.22
MIMAT0005898	hsa-miR-1246	hsa-miR-1246			-4.02
MIMAT0005911	hsa-miR-1260	hsa-miR-1260a	-2.37		-3.98
MIMAT0005922	hsa-miR-1268	hsa-miR-1268a	-5.87	-2.57	
MIMAT0005929	hsa-miR-1275	hsa-miR-1275	-3.52	-3.45	
MIMAT0005939	hsa-miR-1281	hsa-miR-1281	-16.60		-9.92

MIMAT0006764	hsa-miR-320d	hsa-miR-320d	-2.02	1.89	-3.82
MIMAT0006765	hsa-miR-1825	hsa-miR-1825	-18.05		-15.29
MIMAT0007892	hsa-miR-1915	hsa-miR-1915-3p	-4.61		-3.01
MIMAT0009448	hsa-miR-1973	hsa-miR-1973	-13.45		
MIMAT0010313	hsa-miR-762	hsa-miR-762	-5.41		

Supplementary Table 2: logistic regression model including miR-1228-3p and miR-27b-3p urinary levels to predict the diagnosis of DN versus NDRD (upper panel) and DN versus CKD (lower panel).

**DN vs NDRD Variable**	**P value**	**OR**	**95% CI**
**miR-1228-3p (RR/uCr)**	0.01	0.002	0.0006-0.6381
**miR-27b-3p (RR/uCr)**	0.002	0.002	0.001-1.061

**DN vs CKD Variable**	**P value**	**OR**	**95% CI**
**miR-1228-3p (RR/uCr)**	0.002	0.001	0.0007-0.149
**miR-27b-3p (RR/uCr)**	0.007	0.05	0.005-0.552
